# Glycerinated Vaccine Lymph

**Published:** 1897-12-25

**Authors:** 


### The Hospital

A Journal of

The Medical Sciences and Hospital Administration.

Vol. XXIII.—No. 587.] Edited by Sir Henry Burdett, K.C.B., and Solomon C. Smith, M.D., M.R.C.P. [Dec. 25, 1897.

### Glycerinated Vaccine Lymph.

The very remarkable properties of glycerine in preserving the activity of vaccine lymph, and at the same time of destroying any adventitious bacteria which it may either have originally contained, or which may have found access to it during the process of collection, were fully described in the medical officer's supplement to the twenty-fifth report of the Local Government Board, published a few weeks ago. This report has been followed, after an interval of little more than a month, by another in which there is an interesting account of a visit paid by Sir Richard Thorne and Dr. Copeman to the vaccinating stations of Paris, Brussels, Berlin, Dresden, Cologne, and Geneva, in all of which the glycerinated lymph is now being used, generally to the exclusion of the fresh material. The power of glycerine to purify vaccine lymph from adventitious microbes was first announced by Dr. Copeman in 1891, as a discovery reached by a long series of experiments which he had been conducting. Dr. Crookshank had previously succeeded in isolating from lymph, by the method of plate cultivation, an immense number of bacteria, including micrococci, bacilli, torulæ, &c., all of which he recognised as well-known saprophytic bacterial forms, associated, some of them, with processes of suppuration, but none of which could be regarded as the contagium of vaccinia, seeing that not one of them was constantly present in the lymph, whether human or bovine. Dr. Crookshank did not say to what extent methods calculated to exclude extraneous bacteria had formed part of his plan of collection, nor did he attempt to distinguish between the bacteria which were commonly found and those of which the presence might be regarded as accidental. Dr. Copeman, on the other hand, while collecting under the most rigid precautions, found not only the small spore-bearing bacillus, believed by himself and Dr. Klein to be essential to vaccinia, but also three species of micro-organisms, which were present in nearly every specimen of human or of calf lymph that he examined. These were the staphylococcus albus epidermidis, pyogenes aureus, and cereus flavus, the first of which is usually to be found in the upper layers of the healthy skin of unvaccinated persons. In a few cases he found the streptococcus pyogenes, but never the streptococcus of erysipelas, although this microbe is said to have been isolated from vaccine lymph.

It naturally occurred to Dr. Copeman that the exuberant growth of these extraneous organisms might not improbably interfere with, or even supersede, the one which is essential; and he therefore directed his attention to the discovery, if possible, of a means of so treating the lymph as to inhibit the growth of the extraneous organisms without injuring its potency for vaccination. Müller had already discovered, in 1869, that lymph might be diluted with glycerine without suffering any impairment of its activity, and Dr. Stephen Mackenzie had utilised this discovery at the London Hospital, in the small-pox epidemic of 1870-71. It was reserved for Dr. Copeman to discover, not only that the activity of the mixture remained for a long period unimpaired, but that, after it had been kept for a few weeks, all adventitious micro-organisms disappeared.

In the former of the two reports already mentioned, Dr. Copeman gives the details of many experiments; and his paper is illustrated by microphotographs of cultures made from the mixture after various periods of storage. When these cultures had ceased to display a single microbe, the mixture produced good vaccine vesicles in as many as 97 per cent. of the punctures into which it was inserted. The experience of the Continent seems to be absolutely decisive with regard to the soundness of Dr. Copeman's results when tested on the largest scale in practice; and his method not only affords absolute security against the introduction of any other microbe or virus side by side with that of vaccinia, but it also affords the means of almost indefinitely increasing the available supply of lymph. It is calculated in Paris that sufficient diluted lymph for 15,000 insertions may be obtained from a single calf. The material used is the whole internal pulp of the vesicle, to which is added a considerable quantity, sometimes three times its bulk, of pure glycerine, and the compound is then triturated, or ground into a state of perfect admixture of fine division. The whole process is conducted with complete laboratory precautions against the intrusion of pathogenic microbes, and the diluted lymph is at once sealed up in tubes and set aside. Before it is issued, the calf from which the lymph has been taken is slaughtered; and, in the event of the carcass displaying any sign of disease—tuberculous or other—the whole of the lymph obtained from it is destroyed. It would seem that, in this way, parents may obtain an absolute guarantee against any danger to their children, whether real or imaginary, as a consequence of affording them efficient protection against small-pox.

